# A mid-term follow-up of Koutsogiannis’ osteotomy in adult-acquired flatfoot stage II and “early stage III”

**DOI:** 10.1051/sicotj/2017011

**Published:** 2017-03-17

**Authors:** Camilla Arvinius, Elena Manrique, Antonio Urda, Zulema Cardoso, Jose Enrique Galeote, Fernando Marco

**Affiliations:** 1 Hospital Clínico San Carlos Calle Profesor Martín Lagos s/n 28040 Madrid Spain

**Keywords:** Adult-acquired flatfoot, Koutsogiannis’ osteotomy, Medial displacement calcaneal osteotomy, Posterior tibial tendon dysfunction, Stage II, “Early stage III”

## Abstract

*Introduction*: Koutsogiannis’ osteotomy has been widely described to treat adult-acquired flatfoot. However, few articles describe its midterm follow-up. Our aim was to study clinical and radiological outcomes at least one year after surgery and to analyze whether a combined procedure on the medial soft tissue affected these outcomes.

*Methods*: We performed a retrospective study of 30 feet of patients who underwent a Koutsogiannis’ osteotomy due to adult-acquired flatfoot stage II and “early stage III”: a stage III acquired flatfoot without any important structural deformities. The parameters studied were additional medial soft tissue procedures, clinical outcome through the American Orthopaedic Foot and Ankle Society (AOFAS) ankle and midfoot score as well as complications and radiological measurements.

*Results*: Sixteen cases were “early stage III” and 14 stage II. Thirteen patients underwent an associated posterior tibial tendon (PTT) revision: in three cases an end-to-end suture was possible, seven cases needed a FDL transposition, and three underwent synovectomy. Statistically significant improvement was found in the AOFAS score although no significant changes were seen radiologically. No additional benefit was found with the revision of the posterior tibial tendon. As to clinical and radiological results, no differences were found between stage II and “early stage III”. Five cases presented a mild dysesthesia but only one patient needed neurolysis.

*Conclusions*: We consider the Koutsogiannis’ osteotomy to be a safe and effective procedure to reduce pain in patients with stage II and “early stage III” adult-acquired flatfoot.

## Introduction

The clinical manifestation of adult-acquired flatfoot can range from a flexible deformity with normal joint integrity to a rigid arthritic foot [1]. Its deformity is characterized by medial longitudinal arch flattening with an insufficiency of the posteromedial supporting soft tissue. In the acquired flatfoot, osteotomies appear to have a significant role since they permit the restoration of a more normal biomechanics [2]. There are multiple types of osteotomies described, including lateral column lengthening, medial displacement calcaneal osteotomy, and combined double osteotomy technique. The lateral column lengthening has been used extensively for the treatment of flexible flatfoot in combination with medial soft tissue rebalancing procedures but a secondary increase in the calcaneo-cuboid joint pressure has been reported [2, 3]. The medial displacement osteotomy, with or without flexor digitorum tendon transfer, is widely described in the literature as the surgical treatment of acquired flatfoot and although it does not restore the medial column length in all patients, it permits a correction of the deforming valgus force of the Achilles tendon.

Our aim was to study the midterm clinical and radiological results in patients who underwent a Koutsogiannis’ osteotomy due to adult-acquired flatfoot stage II or “early stage III” and to analyze whether a combined procedure on the medial soft tissue affected the outcomes. Even though the major indication for Koutsogiannis’ osteotomy is adult-acquired flatfoot stage II, we also included “early stage III” in older low-demand patients. In these patients a Koutsogiannis’ osteotomy was indicated since we consider it is a less aggressive surgery than the otherwise indicated arthrodesis.

## Methods

We performed a retrospective study of 30 feet in 29 patients (19 female and 10 male) with adult-acquired flatfoot stage II or “early stage III” in old low-demand patients who underwent a Koutsogiannis’ osteotomy between February 2009 and December 2012 at the Department of Traumatology in Hospital Clinico San Carlos, Madrid. The “early stage III” was defined as patients who clinically and radiologically belonged to the stage III where the hindfoot valgus predominated over the other partially reducible deformities. Patients who underwent associated procedures other than a medial soft tissue revision were excluded. The mean age was 56.7 years (range 33.8–78.4) at the moment of surgery with a mean follow-up of 3.2 years (range 1.3–5.1).

The Koutsogiannis’ osteotomy was performed with the patient in lateral decubitus with tourniquet at the level of the thigh. A lateral incision was performed inferoposterior to the peroneal tendons from the upper to the lower surface of the calcaneus, care being taken not to damage the sural nerve. An osteotomy was performed of the posterior calcaneal tuberosity with an oscillating saw and the posterior fragment was displaced medially until its medial border lay in line with the sustentaculum tali, about a one-centimeter displacement. A temporary osteosynthesis was made with one percutaneous posteroanterior K-wire with image intensification along the long axis of the calcaneus, and a second one was used to assure the correction and to avoid any displacement of the posterior tuberosity of the calcaneus. An Acutrak®-screw 6/7(Acumed) was then inserted through the first K-wire and the patients were immobilized with a posterior splint for four weeks.

In patients with a diagnosis of posterior tibial tendon lesion, a revision of this tendon was made changing the patient from lateral decubitus to decubitus prono in order to perform synovectomy, repair or perform a transposition depending on the state of the tendon. The surgical technique consisted of a careful posteromedial ankle incision along the posterior tibial tendon avoiding damage of the neurovascular structures that lies in the proximity of the tendon.

The parameters studied were additional medial soft tissue procedures, clinical outcome through the American Orthopaedic Foot and Ankle Society (AOFAS) ankle and midfoot score as well as complications and radiological measurements in anteroposterior and lateral views (Table 1). All these measurements were made preoperatively and at follow-up.

**Table 1.** Radiological measurements and normal values.

Radiographic view	Angle	Normal values
Lateral	Moreau Costa-Bartani (arch height)	125–130°
Lateral	Meary’s (talo-first metatarsal angle)	0°
Lateral	Talocalcaneal angle (Kite lateral)	25–35°
Lateral	Calcaneal pitch (calcaneal inclination angle)	Low: 10–20°
		Medium: 20–30°
		High: >30°
Lateral	Naviculocuboid overlap (%)	47%
Anteroposterior	Talonavicular coverage	<7°
Anteroposterior	Talar-first metatarsal	−8°

Statistical correlation was analyzed deeming *p* < 0.05 as statistically significant level.

## Results

**Figure 1.**
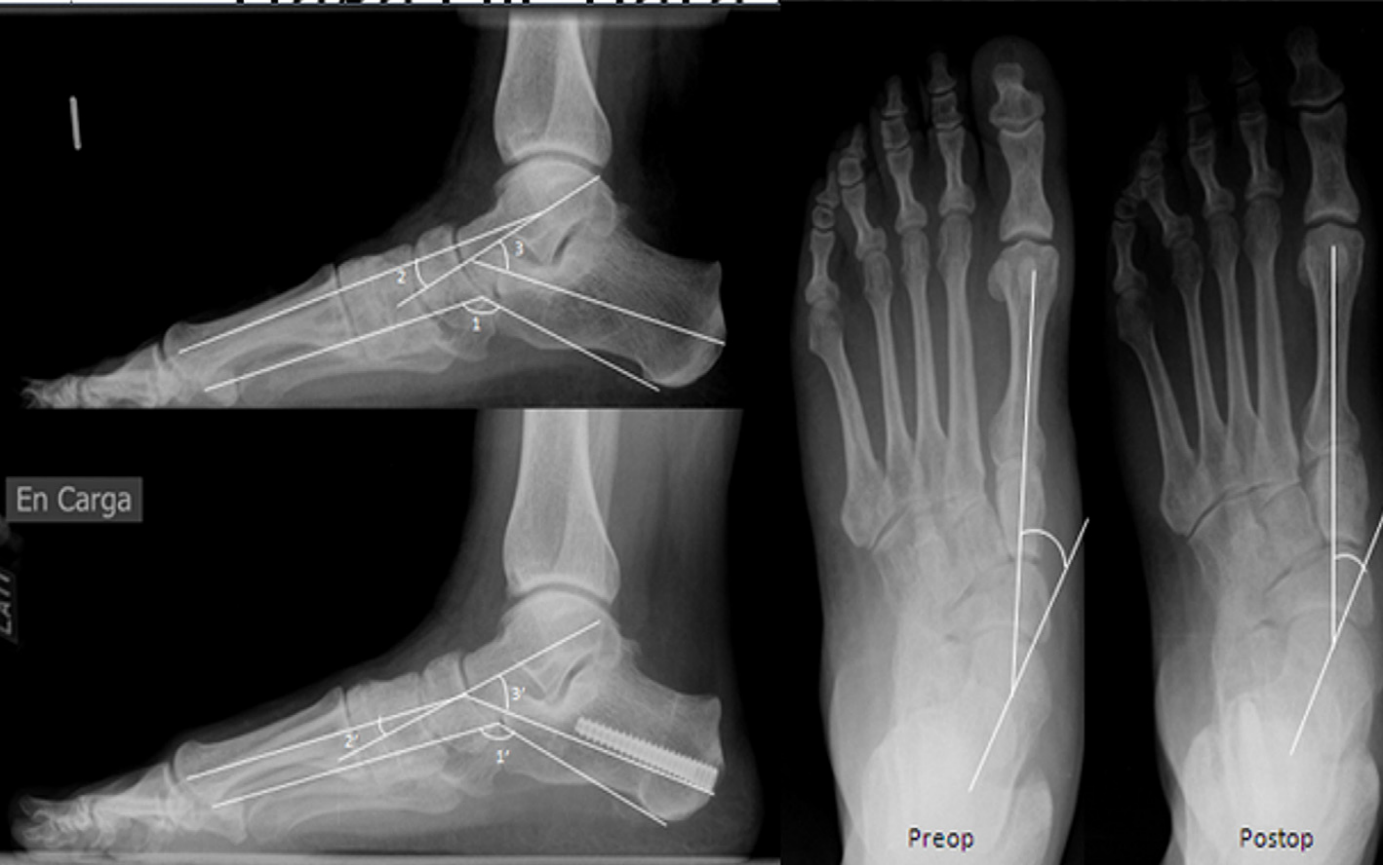
Radiological lateral view of a stage II adult-acquired flatfoot of patients who underwent a Koutsogiannis’ osteotomy: preoperatively and at follow-up 34 months after surgery. Lateral view pre- and postoperatively (’): (1) Moreau Costa-Bartani angle, (2) Meary angle and (3) talocalcaneal angle. Anteroposterior view: talar-first metatarsal angle pre- and postoperatively.

**Figure 2.**
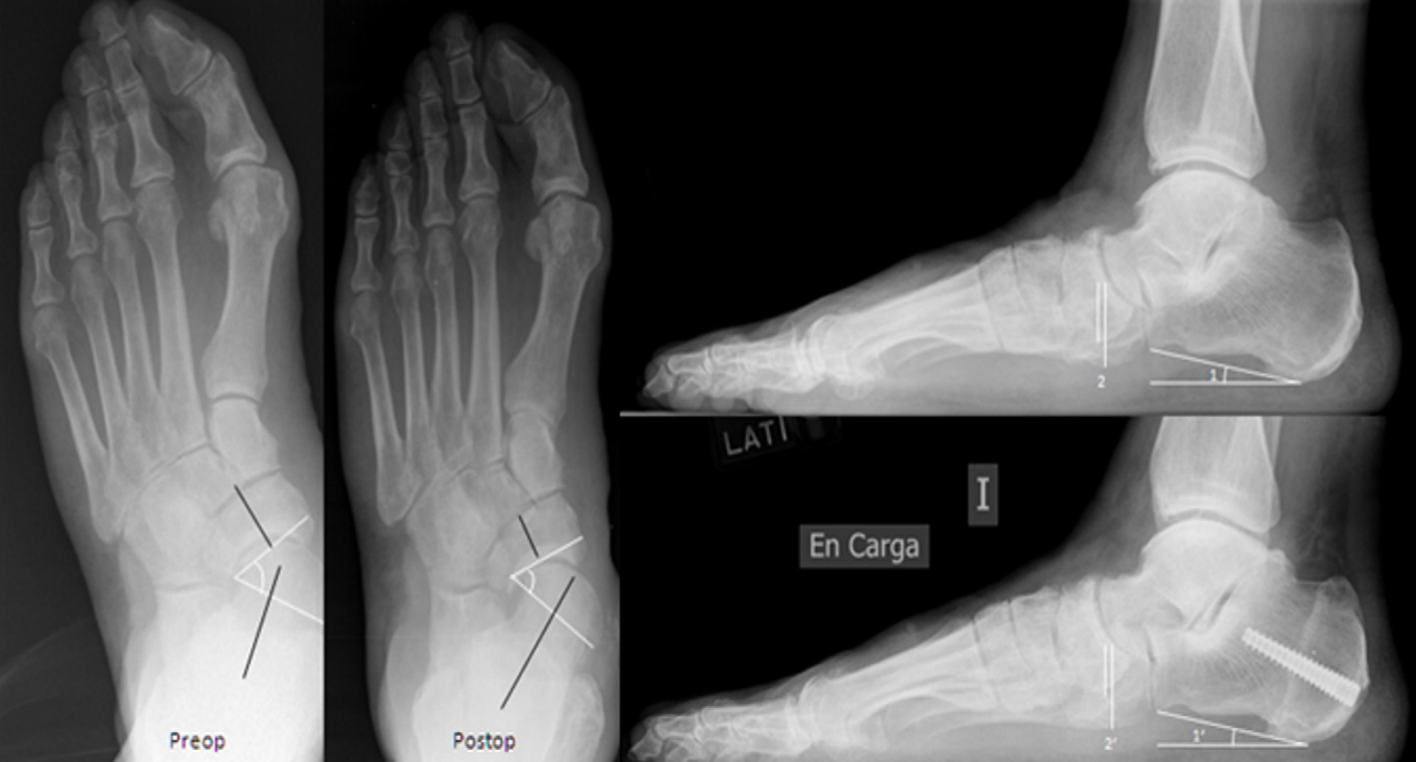
Radiological lateral view of an “early stage III” adult-acquired flatfoot who underwent a Koutsogiannis’ osteotomy preoperatively and at follow-up 24 months after surgery. Lateral view pre- and postoperatively (’): (1) calcaneal pitch and (2) naviculocuboid overlap measured as the overlapped image (short line) divided with the height of the cuboid (long line). Anteroposterior view showing the talonavicular coverage.

Sixteen of the patients presented an adult-acquired flatfoot “early stage III” and 14 were stage II (Figures 1 and 2). All patients underwent a Koutsogiannis’ osteotomy with an Acutrak®-screw 6/7: five cases were 40 mm, 12 were 45 mm, 11 were 50 mm, one of 60 mm, and two of 65 mm.

As to the revision of the posterior tibial tendon: three cases presented a degenerated tendon without rupture, permitting a resection of the degenerated area with a subsequent end-to-end suture. In seven cases it was impossible to repair the posterior tibial tendon and a transposition of the common flexor digitorum tendon was performed, sutured if possible to the posterior tibial tendon and if the previous option was not possible, with an anchor through the navicular bone tunnel. In three cases there was only synovitis without tendon rupture and an isolated posterior tibial synovectomy was performed.

The results of the radiological measurements are shown in Table 2. Better values were observed in all groups, although the difference in the Moreau Costa-Bartani, naviculocuboid overlap, talonavicular coverage, talar-first metatarsal and talocalcaneal angle was greater in the revised PTT group. As to the calcaneal pitch, this angle was not changed in the revision group while it was augmented in the non-revision group. The only statistically significant difference (*p* = 0.044) was found in the correction of the Moreau Costa-Bartani when comparing patients with revision of the PTT with those who underwent an isolated Koutsogiannis’ osteotomy. When comparing radiological measurements between stage II and III or between Koutsogiannis’ osteotomy alone and associated to medial soft tissue procedure no differences were found.

**Table 2.** Radiological measurements in patients who underwent Koutsogiannis’ osteotomy and an associated revision of PTT versus isolated Koutsogiannis’ osteotomy with the *p*-value when comparing both groups.

	Koutsogiannis’+associated PTT revision			Isolated Koutsogiannis’			
	Preop. values	Postop. values	Correction	Preop. values	Postop. values	Correction	*p*-value
Lateral view							
Moreau Costa-Bartani angle Arch height (°)	149.71 (133–171)	141.67 (124–161)	6.17 (−1–15)	145.43 (129–165)	146.14 (133–162)	0.48 (−23–15)	0.044
Meary angle Talar-first metatarsal (°)	7.57 (1–24)	8.58 (5–14.7)	−0.08 (−4.8–9.3)	8.38 (0–19.9)	8.00 (0–18.3)	−0.25 (−8.3–9.3)	0.925
Talocalcaneal (°)	29.05 (15–43)	32.71 (21-42)	5.62 (−12–14)	32.95 (14.6–52)	36.01 (9–53)	3.22 (−17.7–17)	0.498
Calcaneal pitch (°)	7.88 (0–17.2)	8 (−3–17)	−0.3 (−5–8.7)	13.41 (−3–25)	10.69 (−6–22.2)	2.48 (−6.5–21.8)	0.334
Naviculocuboid overlap (%)	46.17 (18–72)	29.37 (21.3–46)	−15.56 (−4.7–44.8)	39.51 (0.4–73)	32.65 (11.2–67)	−8.21 (−17.8–44.8)	0.240
AP view							
Talonavicular coverage (°)	38.22 (20–64)	45.8 (25–63)	−7.68 (−29–19)	44.5 (10–92)	42.19 (3–88)	−4.92 (−49–21)	0.767
Talar-first metatarsal (°)	14.83 (9–20)	20.26 (14.5–26.9)	−6.83 (−13.9–0.6)	18.94 (3–33)	16.91 (1.4–37)	−0.68 (−32–20)	0.230

As to the clinical outcomes (Table 3) statistically significant difference was found showing better postoperative values in both groups (*p* = 0.038 in revision vs. 0.032 in non-revision) however, no differences between groups were found.

**Table 3.** Clinical outcomes with AOFAS ankle and midfoot score in patients who underwent Koutsogiannis’ osteotomy and an associated PTT revision versus isolated Koutsogiannis’ osteotomy with the *p*-value when comparing both groups.

	Koutsogiannis’+associated PTT revision			Isolated Koutsogiannis’			
AOFAS	Preop.	Postop.	Correction	Preop.	Posto.	Correction	*p*-value
Pain	17.65	34.29	16.64	16.34	31.30	14.96	0.303
Function	19.23	46.71	27.48	20.78	43.91	23.13	0.966
Alignment	9.29	9.29	0	8.04	8.04	0	0.413
Total	46.17	90.29	44.12	45.16	83.25	38.09	0.172

As to the complications, these were registered in five patients: all presented a mild dysesthesia of the sural territory but only 1 patient needed a neurolysis. No infections or nonunions were detected. The complications were not statistically significant between groups.

## Discussion

The osteotomies are an essential part of the surgical treatment of the adult-acquired flatfoot since they modify the biomechanics of the hindfoot in different ways according to their relation to the posterior subtalar facet [4]. An anterior calcaneal osteotomy corrects deformities in the transverse plane (forefoot abduction) whereas a posterior tuberosity osteotomy results in varization of the calcaneus and a correction in the frontal plane [2]. Anatomically, in a flexible flatfoot the calcaneus is in a valgus position while the talus is rotated medially and inferiorly due to changes in the subtalar joint; it is tilted medially and lies lower than normal [5]. It is unknown whether the pre-existing flatfoot predisposes the development of posterior tibial tendon dysfunction (PTTD) or if a PTTD (due to direct traumatic injuries, systemic diseases, etc.) leads to a flatfoot [6]. Hence, the alignment of the bones in the medial column is lost producing a subluxation of the talonavicular joint [5]. An abduction of the midtarsal joint can be found along with a pronated forefoot. The alteration of the normal anatomy produces a clinical deformity consisting of flattening of the longitudinal arch and valgus of the hindfoot. Thereby, the weight-bearing of the foot is transmitted medial to the calcaneus through the talus.

As to the radiographic measurements there are no gold standards for foot deformities. The talo-first metatarsal angle is a valuable radiographic identifier of adult-acquired flatfoot and the calcaneal pitch has a good interobserver reliability for analyzing the deformity [7]. However, in our study this angle was the only one that did not show concordance with the other results since all the other parameters showed better results postoperatively in both groups. Lee et al. [8] studied the reliability and validity of the different angles in anteroposterior and lateral weight-bearing radiographs in hindfoot varus and valgus. The naviculocuboid overlap, the anteroposterior talus-first metatarsal angle, and the talonavicular coverage angle were found to be reliable and valid methods for discriminating these deformities. In our study all the previously stated radiological parameters were symmetrical supporting the results from Lee et al. These results are thought to be useful, especially when attempting to objectively quantify the severity of the foot deformity since the diagnostic criteria for a foot deformity generally are clinical and depend on visual inspection.

According to Guha and Perera [4] the choice of the type of osteotomy depends on the plane of the dominant deformity. Clinically, the patient presents a marked hindfoot valgus and the medializing osteotomy of the calcaneal tuberosity is used to biomechanically displace the calcaneal weight-bearing axis medially thereby aligning it with the tibial axis to restore the function of the gastrosoleus as a heel invertor. As to the lateral column lengthening, described as the primary procedure of the flexible flatfoot, some authors [7] are limiting its indications due to the high rate of complications such as an increase in the calcaneo-cuboid joint pressure or lateral column pain. We consider that the lateral column lengthening could still be indicated in patients with flexible feet where a forefoot abduction is found and that it could be made at the same surgical time as the Koutsogiannis’ osteotomy.

Koutsogiannis’ osteotomy was first described in 1893 by Gleich [9] who displaced the posterior calcaneal fragment in an anteroinferomedial direction in an attempt to restore a normal calcaneal pitch. Despite various modifications of this procedure over the years, the underlying principle of the correction remains the same [6]. In 1971 Koutsogiannis [5] described a lateral approach and a medializing calcaneal osteotomy that permitted a successful correction of the valgus deformity and longitudinal arch. Although no radiological differences were found when comparing the preoperative with the postoperative value in our study we agree with many other articles [1–7] that describe the medializing calcaneal osteotomy as an effective procedure to relieve pain in patients with a flexible flatfoot. In our experience, the clinical changes are generally seen six to nine months after surgery, possibly due to a certain delay between the osteotomy and the final biomechanical effect of the Achilles tendon. Although there is no validated protocol for choosing one procedure over another (such as lateral column lengthening, medial displacement osteotomy, or combined double osteotomy technique), the flexible deformity should be treated appropriately to preserve function and minimize the surgical morbidity that accompanies a fixed flatfoot deformity [2, 4, 6].

Some authors [2, 3] advocate the concomitant reconstruction of the posterior tibial tendon or flexor digitorum tendon transfer since it permits the osteotomy to act as a double tendon transfer with the flexor digitorum longus (FDL) to aid foot inversion. The PTT is explored and, if it is not severely degenerated a reparation is performed by excision of the degenerated areas [6, 7, 10]. In cases where a reparation is not possible, a flexor digitorum longus tendon-to-medial cuneiform or navicular tendon transfer with debridement or removal of the posterior tendon should be performed. Other authors [7] recommend that the reconstruction of soft tissue is performed last and only when necessary or even that it should be avoided. A biomechanical analysis [11] showed that the FDL transfer did little to reduce the load on the medial column, whereas the medial column osteotomy itself greatly reduced the load. DiDomenico et al. [12] supported this in a clinical retrospective study since they noticed a significant structural realignment without the use of FDL transfer. As to clinical and radiological outcomes, we were not able to find any statistical significant differences between the patients who underwent revision of the PTT and those who did not except for the Moreau Costa-Bartani angle.

In the more severe flatfoot the triple arthrodesis has traditionally been the procedure of choice [13–15]. It has proven to be a predictable procedure with universally good results and high patient satisfaction [13]. Nonetheless, complications have been reported such as degeneration of surrounding joints, inadequate realignment in cases with severe transverse plane deformity, increased risk of forefoot varus deformity, and lateral wound problems. Some authors [14] refer that they prefer double arthrodesis when a severe subluxation of the TNJ is found on the AP radiograph. We found no clinical nor radiological significant differences between stage II and “early stage III”. Therefore, we consider the Koutsogiannis’ osteotomy as an option for older low-demand patients with a stage III flatfoot without great deformities since it is a less aggressive method than a triple arthrodesis.

In our study we excluded patients who underwent adjunct procedures other than medial soft tissue procedures. However, we agree with the literature reviewed [13–15] that it is important to consider the necessity of adjunct procedures such as gastrocnemius recession or medial displacement osteotomy of the calcaneus in persistent ankle valgus. A medial column stabilization could also be considered either in the form of naviculocuneiform arthrodesis or first tarsometatarsal arthrodesis.

In our study we were not able to detect any differences, especially as to the AOFAS score, between the patients who underwent only osteotomy or those who underwent both osteotomy and revision and therefore tend not to revise the PTT tendon after this study. However, we indicated a Koutsogiannis’ osteotomy not only in grade II patients but some that radiologically corresponded to a grade III without any significant deformities except for the proper stage III deformities. A Koutsogiannis’ osteotomy was indicated in these older low-demand cases since we consider it a less aggressive surgery than a triple arthrodesis and we found these patients to evolve as satisfactorily as grade II. We therefore consider the Koutsogiannis’ osteotomy to be a safe and effective procedure to reduce pain in patients with stage II and older low-demand patients with “early stage III” adult-acquired flatfoot.

## Conflict of interest

The authors certify that he or she has no financial conflict of interest (e.g., consultancies, stock ownership, equity interest, patent/licensing arrangements, etc.) in connection with this article.

## References

[1] Giza E, Cush G, Schon L (2007) The flexible flatfoot in adult. Foot Ankle Clin 12(2), 251–271. 17561199 10.1016/j.fcl.2007.03.008

[2] Mosier-LaClair S, Pomeroy G, Manoli A 2nd (2001) Operative treatment of the difficult stage 2 adult acquired flatfoot deformity. Foot Ankle Clin 6(1), 95–119. 11385931 10.1016/s1083-7515(03)00083-4

[3] Hintermann B, Valderrabano V, Kundert H (1999) Lengthening of the lateral column and reconstruction of the medial soft tissue for treatment of acquired flatfoot deformity associated with insufficiency of the posterior tibial tendon. Foot Ankle Int 20(10), 622–629. 10540992 10.1177/107110079902001002

[4] Guha A, Perera A (2012) Calcaneal osteotomy in the treatment of adult acquired flatfoot deformity. Foot Ankle Clin 17(2), 247–258. 22541523 10.1016/j.fcl.2012.02.003

[5] Koutsogiannis E (1971) Treatment of mobile flat foot by displacement osteotomy of the calcaneus. J Bone Joint Surg Br 53(1), 96–100. 5578768

[6] Hix J, Kim C, Mendicino R, Saltrick K, Catanzariti A (2007) Calcaneal osteotomies for the treatment of adult-acquired flatfoot. Clin Podiatr Med Surg 24(4), 699–719. 17908638 10.1016/j.cpm.2007.07.002

[7] Hentges M, Moore K, Catanzariti A, Derner R (2014) Procedure selection for the flexible adult acquired flatfoot deformity. Clin Podiatr Med Surg 31(3), 363–379. 24980927 10.1016/j.cpm.2014.03.003

[8] Lee K, Chung C, Park M, Lee S, Cho J, Choi I (2010) Reliability and validity of radiographic measurements in hindfoot varus and valgus. J Bone Joint Surg Am 92(13), 2319–2327. 20926727 10.2106/JBJS.I.01150

[9] Silver C, Simon S, Spindell E, Litchman H, Scala M (1967) Calcaneal osteotomy for valgus and varus deformities of the foot in cerebral palsy. A preliminar report on twenty-seven operations. J Bone Joint Surg Am 49(2), 232–246. 5335171

[10] Myerson M, Badekas A, Schon L (2004) Treatment of stage II posterior tibial tendon rupture with flexor digitorum longus tendon transfer and calcaneal osteotomy. Foot Ankle Int 25(7), 445–450. 15319100 10.1177/107110070402500701

[11] Arangio G, Salathe E (2009) A biomechanical analysis of posterior tibial tendon dysfunction, medial displacement calcaneal osteotomy and flexor digitorum longus transfer in adult acquired flat foot. Clin Biomech 24(4), 385–390. 10.1016/j.clinbiomech.2009.01.009 19272682

[12] DiDomenico L, Stein D, Wargo-Dorsey M (2011) Treatment of posterior tibial tendon dysfunction with flexor digitorum tendon transfer: a retrospective study of 34 patients. J Foot Ankle Surg 50(3), 293–298. 21397524 10.1053/j.jfas.2010.12.011

[13] Catanzariti AR, Dix BT, Richardson PE, Mendicino RW (2014) Triple arthrodesis for adult acquired flatfoot. Clin Podiatr Med Surg 31(3), 415–433. 24980931 10.1016/j.cpm.2014.03.004

[14] Catanzariti AR, Adeleke AT (2014) Double arthrodesis through a medial approach for end-stage adult-acquired flatfoot. Clin Podiatr Med Surg 31(3), 435–444. 24980932 10.1016/j.cpm.2014.04.001

[15] Johnson J, Yu J (2006) Arthrodesis techniques in the management of stage II and III acquired adult flatfoot deformity. Instr Course Lect 55, 531–542. 16958486

